# Interleukin-17 impedes Schwann cell-mediated myelination

**DOI:** 10.1186/1742-2094-11-63

**Published:** 2014-03-29

**Authors:** Mark Stettner, Birthe Lohmann, Kathleen Wolffram, Jan-Philipp Weinberger, Thomas Dehmel, Hans-Peter Hartung, Anne K Mausberg, Bernd C Kieseier

**Affiliations:** 1 Department of Neurology, Medical Faculty, Research Group for Clinical and Experimental Neuroimmunology, Heinrich-Heine-University, Moorenstraße 5, 40225 Düsseldorf, Germany

**Keywords:** dorsal root ganglia, myelin, IL-17, Guillain-Barré syndrome, inflammatory neuropathy

## Abstract

**Background:**

Pro-inflammatory cytokines are known to have deleterious effects on Schwann cells (SCs). Interleukin 17 (IL-17) is a potent pro-inflammatory cytokine that exhibits relevant effects during inflammation in the peripheral nervous system (PNS), and IL-17-secreting cells have been reported within the endoneurium in proximity to the SCs.

**Methods:**

Here, we analyzed the effects of IL-17 on myelination and the immunological properties of SCs. Dorsal root ganglia (DRG) co-cultures containing neurons and SCs from BL6 mice were used to define the impact of IL-17 on myelination and on SC differentiation; primary SCs were analyzed for RNA and protein expression to define the putative immunological alignment of the SCs.

**Results:**

SCs were found to functionally express the IL-17 receptors A and B. In DRG cultures, stimulation with IL-17 resulted in reduced myelin synthesis, while pro-myelin gene expression was suppressed at the mRNA level. Neuronal outgrowth and SC viability, as well as structural myelin formation, remained unaffected. Co-cultures exhibited SC-relevant pro-inflammatory markers, such as matrix metalloproteinase 9 and SCs significantly increased the expression of the major histocompatibility complex (MHC) I and exhibited a slight, nonsignificant increase in expression of MHCII, and a transporter associated with antigen presentation (TAP) II molecules relevant for antigen processing and presentation.

**Conclusions:**

IL-17 may act as a myelin-suppressive mediator in the peripheral nerve, directly propagating SC-mediated demyelination, paralleled by an inflammatory alignment of the SCs. Further analyses are warranted to elucidate the role of IL-17 during inflammation in the PNS *in vivo*, which could be useful in the development of target therapies.

## Background

Immune-mediated neuropathies represent a heterogeneous group of mainly demyelinating conditions, including chronic inflammatory demyelinating polyneuropathy (CIDP) and Guillain-Barré syndrome (GBS). Both conditions are caused by an autoimmune response to peripheral nerve antigens leading to inflammation, followed by glial and neuronal damage. The underlying molecular mechanism of these diseases still remains mainly unclear.

Interleukin (IL)-17 exhibits relevant effects during inflammation in the peripheral nervous system (PNS) [1]. In addition, in chronic constriction injury of the sciatic nerve, IL-17^+^ T cells have been detected in the endoneurium, and contribute to myelin damage [2]; furthermore, IL-17 mediates a role in ensuing neuropathic pain [3].

IL-17 is a mainly pro-inflammatory cytokine, which is expressed by CD4^+^ effector T helper cells (Th17 lineage), as well as by other immune cells, such as neutrophils and eosinophils [4-7].

Secretion of IL-17 by peripheral blood mononuclear cells (PBMCs) in CIDP patients with disease activity was found to be elevated compared with controls, and also elevated in active CIDP patients compared with those in remission; other interleukins did not show this correlation [8]. Even in the absence of cerebrospinal fluid (CSF) pleocytosis, IL-17 was found to be elevated in the CSF of CIDP patients, showing a strong positive correlation with CSF protein concentration [9]. Chi *et al*. detected an increase in Th17^+^ cell populations in the CSF of active CIDP patients [10]. These results suggest an involvement of IL-17 during the active and demyelinating phase of the disease.

In an animal model of GBS, IL-17 was shown to be present in rat sciatic nerves after induction of experimental autoimmune neuritis [11]; IL-17 seemed to play a role [12], particularly in the induction phase of the condition. Additionally, CSF and plasma levels of IL-17 and IL-22 were found to be elevated in GBS patients compared with healthy controls, and the IL-17 and the related IL-22 levels in the CSF correlated with the GBS disability scale scores [13]. All these studies claim a crucial role for IL-17 but fail to decipher the molecular mechanism of IL-17 in the inflamed peripheral nerve.

The fact that IL-17 is present in the inflamed peripheral nerve and that the corresponding receptor (IL-17 receptor (IL-17R)), is ubiquitously expressed [14] may suggest a direct effect of IL-17 on Schwann cells (SCs). SCs play the undisputable leading role in myelinating peripheral nerves as well as in remyelination after injury [15], thereby ensuring signal transmission and maintenance of neuronal homeostasis. Besides these properties, SCs orchestrate PNS immunology, similar to non-myelinating astrocytes in the central nervous system [16]. To this end, SCs possess the ability to secrete cytokines and modify and express antigens on the major histocompatibility complex (MHC) on their surface [17,18]. As immune-qualified glia, SCs functionally express high levels of toll-like receptors (TLRs), mainly TLR3 and TLR4, which respond to their respective ligands [19]. Various inflammatory or pro-inflammatory mediators are reported to modulate SC homeostasis, such as interleukins, inducible nitric oxide synthase (iNOS), cyclo-oxygenase-2 (COX-2), and matrix metalloproteinases (MMPs), some of them in an autocrine or paracrine manner, as a particular response to the surrounding environment [20-24]. As such, the inflammatory mediator tumor necrosis factor α (TNF-α) promotes phenotype reversion of mature SCs to immature SCs [25-27].

These results are most likely just fragments towards our understanding of the complex signaling of SC differentiation to myelination or an immune-active phenotype. In order to define the impact of IL-17 on SC differentiation and to detect a putative immunological alignment of the SCs, we analyzed the effect of IL-17 on myelinating dorsal root ganglia (DRG) co-cultures and on purified SC cultures.

## Methods

### Primary Schwann cell cultures

Mouse SCs (mSCs) were prepared using a modified Brockes method [28]. Cells were purified and cultured as described before [29]. Briefly, sciatic nerves were dissected from neonatal (postnatal day 3 (P3)) C57BL/6 mice and digested with 0.05% collagenase (Worthington, Lakewood, NJ, USA) and 0.125% trypsin (Merck, Darmstadt, Germany). Cells were plated in poly-D-lysine (PDL)-coated (Sigma-Aldrich Corp., St. Louis, MO, USA) cell culture dishes (Greiner Bio-One GmbH, Frickenhausen, Germany) with Dulbecco’s Modified Eagle’s Medium (DMEM; Invitrogen Corp., Carlsbad, CA, USA) containing 10% horse serum (HS; Invitrogen Corp., Carlsbad, CA, USA), 4 mM L-glutamine ((Glut) Invitrogen Corp., Carlsbad, CA, USA), 2 ng/mL human heregulin β-1 (Cell Sciences, Canton, MA, USA), 0.5 μM forskolin (FKL) (Sigma-Aldrich Corp., St. Louis, MO, USA) and 100 IU/mL penicillin/streptomycin (PS; Invitrogen Corp., Carlsbad, CA, USA). For complement lysis, cells were washed with Hank’s Balanced Salt Solution (HBSS; Invitrogen Corp., Carlsbad, CA, USA) containing 4-(2-hydroxyethyl)-1-piperazine ethane sulfonic acid (HEPES; Invitrogen Corp., Carlsbad, CA, USA) and subsequently incubated with DMEM, containing HEPES, HS, Glut, P/S and anti-thymidine 1.2 antibody (AbD Serotec, Kidlington, UK). After 15 min at 37°C, rabbit complement (Cedarlane Laboratories Inc., Burlington, NC, USA) was added and incubated for 2 h at room temperature (RT). Cells were washed twice using HBSS containing HEPES and cultured on PDL-coated culture dishes in DMEM medium, containing 10% HS (Invitrogen Corp., Carlsbad, CA, USA), 4 mM Glut (Invitrogen Corp., Carlsbad, CA, USA), 2 ng/mL human heregulin β-1 (Cell Sciences, Canton, MA, USA), 0.5 μM forskolin (Sigma-Aldrich Corp., St. Louis, MO, USA), 20 μg/mL pituitary extract bovine (PEB, Merck Millipore, Darmstadt, Germany), 10 ng/mL recombinant human fibroblast growth factor (Biomol GmbH, Hamburg, Germany), 100 IU/mL P/S (Invitrogen Corp., Carlsbad, CA, USA) and 1:4 mouse DRG supernatants; the medium was renewed every third or second day.

Preparation of rat SCs (rSCs) was performed using the modified Brockes method [28]. Sciatic nerves were dissected from neonatal (P3) Wistar rats, digested with 0.1% collagenase (Worthington, Lakewood, NJ, USA) and 0.25% trypsin (Invitrogen Corp., Carlsbad, CA, USA), and cells were finally plated with DMEM containing 10% fetal calf serum (FCS). Cultures were treated with two cycles of 10 μM cytosine arabinoside to reduce fibroblasts, followed by complement lysis with anti-thymidine 1.1 antibodies. Cultures reached a final purity of more than 95% and were maintained in DMEM Gibco 3185 (Invitrogen Corp., Carlsbad, CA, USA) with 10% FCS, 100 IU/mL P/S, 2 mM Glut, and 1 μL/mL FKL on PDL-coated culture dishes.

### Preparation of dorsal root ganglia

DRGs were prepared from embryonic day 15 (E15) C57BL/6 mice (BL6) by opening the cutis and subcutis along the spine and removing the spinal cord. DRG were collected, centrifuged and resuspended [29,30]. Twenty-four well plates (Greiner Bio-One AG, Frickenhausen, Germany) were pre-coated twice with collagen type I (Becton Dickinson AG, Franklin Lakes, New Jersey, USA) and 0.02 N acetic acid (1:6) (Carl Roth GmbH, Karlsruhe, Germany), before plating the ganglia cells. The DRG cultures were kept in neurobasal medium for 7 days, DMEM medium containing (BioWhittacker, Lonza Group AG, Basel, Switzerland), 2 mM Glut (Invitrogen Corp., Carlsbad, CA, USA) 10% HS (Invitrogen Corp., Carlsbad, CA, USA), 100 IU/L P/S (Invitrogen Corp., Carlsbad, CA, USA), 100 ng/mL nerve growth factor (NGF; Sigma-Aldrich corp., St. Louis, MO, USA), and 4 g/L glucose (Sigma-Aldrich Corp., St. Louis, MO, USA). After 1 week of culture, neurobasal medium was exchanged for myelination media containing minimal essential media (MEM, Invitrogen Corp., Carlsbad, CA, USA), 20 μg/mL PEB (Merck Millipore, Darmstadt, Germany), 50 mg/L L-ascorbic acid (AA, Sigma-Aldrich Corp., St. Louis, MO, USA), 0.5 μM FKL (Sigma-Aldrich Corp., St. Louis, MO, USA), 2 mM L-glut (Invitrogen Corp., Carlsbad, CA, USA), 5% HS (Invitrogen Corp., Carlsbad, CA, USA), 1× N2-supplement (N2; Invitrogen Corp., Carlsbad, CA, USA), 4 g/L D-(+)-glucose 10% (Sigma-Aldrich Corp., St. Louis, MO, USA), and 50 ng/mL NGF (Sigma-Aldrich Corp., St. Louis, MO, USA). The culture myelination medium was renewed every 3 to 4 days. Cultures were kept for 28 days *in vitro* and treated as indicated from the sixth day after explantation until fixation, followed by staining.

### Immunocytochemistry

For immunocytochemistry, cells grown on glass cover slips were initially washed with phosphate-buffered saline solution (PBS) and fixed with 4% paraformaldehyde (PFA; Merck, Darmstadt, Germany) for 30 min for NF-L (neurofilament L) and 10 min for IL-17 receptor (IL-17R), following another washing step with PBS containing 1% bovine serum albumin (BSA; Sigma-Aldrich Corp., St. Louis, MO, USA). Samples were blocked using PBS-based blocking solution containing 10% (NF-L) or 4% (IL-17R) natural goat serum (NGS, DAKO, Hamburg, Germany) and 0.1% (NF-L) or 0.2% (IL-17R) Triton X-100 (Merck, Darmstadt, Germany) for 30 min at RT. We used primary antibodies against IL-17 receptor A (IL-17R A; Abcam, Cambridge, UK), IL-17 receptor B (IL-17R B; Abcam, Cambridge, UK), and rabbit anti-NF-L (Millipore, Billerica, MA, USA), each diluted 1:400. Furthermore, antibodies against MHCI (1:750, mouse monoclonal antibody; Novus Biologicals, Littleton, CO, USA), MHCII (1:50, mouse monoclonal antibody; AbD Serotec Kidlington, UK) and transporter associated with antigen presentation (TAP) II (1:200, rabbit polyclonal; Bioss, Woburn, MA, USA) were used.

Primary antibodies were diluted in PBS, containing 0.1% Triton (0.05% for the MHCI antibody), 10% NGS, and for MHCII, an additional 0.25% BSA. Cells were incubated for 1 hour at 37°C (overnight at 4°C for NF-L). After three washing cycles with PBS, the secondary antibody was applied for 1 hour at RT. The following secondary antibodies were used: Alexa Fluor™ 594 goat anti-rabbit, Alexa Fluor™ 594 mouse anti-rabbit, Alexa Fluor™ 594 goat anti-rabbit (Invitrogen Corp., Carlsbad, CA, USA), 1:200 diluted in PBS and 1% BSA (Sigma-Aldrich Corp., St. Louis, MO, USA) and for NF-L 1:400 diluted in antibody diluent, followed by three washing cycles with PBS. Samples were embedded in 4′,6-diamidine-2′-phenylindole dihydrochloride (DAPI) containing mounting medium (Vectashield™, Vector Laboratories Inc., Burlingame, CA, USA) and analyzed with an upright fluorescence microscope (Nikon Eclipse TE200, Nikon AG, Tokyo, Japan and Axioplan 2 Imaging, Zeiss, Oberkochen, Germany).

### Real-time polymerase chain reaction

Total cellular RNA was extracted using an RNeasy™ Mini Kit (Qiagen, Hilden, Germany) and quantified by NanoDrop-1000 (PEQLAB, Erlangen, Germany). Cells were washed twice with PBS and detached with buffer RLT. Total RNA (400 ng) was applied as matrix for cDNA synthesis using TaqMan™ Reverse Transcription Reagents (Applied Biosystems, Foster City, CA, USA) and High-Capacity cDNA Reverse Transcription Kit (Applied Biosystems, Foster City, CA, USA) in accordance with the manufacturer’s protocol (10 min at 25°C, 120 min at 37°C, and 5 min at 85°C). For subsequent real-time polymerase chain reaction (rtPCR) the thermal cycler (AbiPrism7000, Foster City, CA, USA) was set to run for 2 min at 50°C, 10 min at 95°C, 40 cycles at 95°C for 15 sec, and 1 min at 60°C. Power SYBR Green PCR Master Mix (Applied Biosystems, Foster City, CA, USA) and TaqMan™ Universal PCR Mastermix (Applied Biosystems, Foster City, CA, USA) were used. cDNA was inserted for amplification at a final concentration of 0.6 μM for each primer. rtPCR was followed by a melting curve analysis. Overall, the experiments were performed with the housekeeping genes 18S (rRNA probe dye, VIC-MGB, Applied Biosystems, Foster City, CA, USA) and glyceraldehyde-3-phosphate dehydrogenase (GAPDH) to calculate ∆∆ct and shown as expression correlated to housekeeping gene and control expression [31]. cDNA was amplified with the following primers: for IL-17A, 5′-TGG GAT CTG TCA TCG TGC T-3′ and 5′-ATC ACC ATG TTT CTC TTG ATC G-3′; for IL-17B: 5′-GGA CAG CCC TTC TTT GTC TG-3′ and 5′-TGC TTT TTA TAT TTC ATT ACG TGG TT-3′; for IL-17C, 5′-CCA CCC CAA CCT CTG TGT-3′ and 5′-CAA GGA GTC AGC CCA CGA-3′; for P0, 5′-ACC TTC AAG GAG CGC ATC C-3′ and 5′- GCC ATC CTT CCA GCT AGG GT -3′; for KROX-20, 5′-CTG GGC AAA GGA CCT TGA TG-3′ and 5′-GTC CGT GAG AAG GTG GGA CA-3′. Four independent experiments were performed, and for each experiment, three PCR runs, each in triplicate, were analyzed.

### Sudan staining

Cultures were stained with Sudan black dye to assess *in vitro* myelination [32]. Sudan staining has been approved before as an efficient and reliable method for myelin quantification in DRG co-cultures [29]. After washing twice with PBS, cells were fixed for 1 hour using 4% PFA (Sigma-Aldrich Corp., St. Louis, MO, USA), then washed twice again with PBS and treated with 0.1% osmium tetroxide (Sigma-Aldrich corp., St. Louis, MO, USA) for 1 hour. After sequential ethanol treatment (25%, 50%, 70%, each for 5 min), myelin was stained using 0.5% Sudan black (Flukan, Zurich, Switzerland) dissolved in 70% ethanol for 1 hour, followed by treatment with ethanol at decreasing concentrations (70%, 50%, 25% each for 1 min). Myelin was analyzed after Sudan-Black staining, using an upright microscope (Nikon Eclipse TE200, Nikon AG, Tokyo, Japan). The complete cell layer was recorded using a 20x objective, and individual pictures were reconstructed using Adobe Photoshop (version 8.0, Adobe Systems Incorporated, Delaware, USA). Quantification was performed by counting the number of internodes and correlating them to the number of neurons within the cultures. Counting was performed using ImageJ (version 1.41o, National Institutes of Health, USA). To account for inter-experimental variability of the quotient, data are shown on an ordinal scale and normalized to 100%.

### Electron microscopy

DRG co-cultures were grown on collagen-coated plastic dishes, washed with PBS, and fixed for 12 h with 1% glutaraldehyde (Serva GmbH, Heidelberg, Germany) and 6% tannin (Merck KGaA, Darmstadt, Germany) in 0.1 M cacodylate buffer (Merck KGaA, Darmstadt, Germany), followed by postfixation in 1% osmium tetroxide (Sigma-Aldrich Corp., St. Louis, MO, USA) for 1 h, dehydrated using a series of alcohol, and finally embedded in Epon. Ultrathin sections were prepared from regions of interest and analyzed by electron microscopy (EM 910; Carl Zeiss, Oberkochen, Germany).

### Viability assay

A CellTiter-Blue™ Assay (Promega, Madison, WI, USA) was performed to measure cell viability after IL-17 stimulation for 10 days *in vitro*. Analysis was performed with cells grown in 96-well plates (Greiner Bio-One AG, Frickenhausen, Germany), 12 samples per concentration and three independent experiments were performed. The CellTiter-Blue™ reagent was added directly to cells cultured in serum-supplemented medium. After an incubation period of 1 to 3 h, spectral data (579Ex/584Em) were recorded using a GENios-Pro reader (LI-COR Biosciences, Bad Homburg, Germany) and analyzed statistically.

### MMP-9 and MMP-2 activity

Gelatinase activity was measured by sodium dodecyl sulfate-polyacrylamide gel electrophoresis (SDS/PAGE) as described before [32,33]. A 12 μL amount of culture supernatants was incubated with Novex™ Tris-glycine sodium dodecyl sulfate sample buffer and applied to a 10% polyacrylamide gel (Bio-Rad Laboratories, Hercules, CA, USA) containing 0.1% sodium dodecyl sulfate (Sigma-Aldrich Corp., St. Louis, MO, USA) and 0.1% gelatin from porcine skin (Sigma-Aldrich Corp., St. Louis, MO, USA); the stacking gels were 5% polyacrylamide gels. After electrophoresis and washing, incubation in Novex™ Zymogram developing buffer (Invitrogen Corp., Carlsbad, CA, USA) for 20 h at RT was followed by staining for 3 h in 30% methanol (Merck, Darmstadt, Germany), 10% acetic acid (Carl Roth GmbH, Karlsruhe, Germany) containing 0.5% Coomassie brilliant blue (Sigma-Aldrich Corp., St. Louis, MO, USA). Finally, gels were destained using staining buffer without dye. Gelatinase activity was quantified using densitometry of unstained bands representing the areas of gelatin digestion. Densitometry was performed using ImageJ, a public domain image processing program (version 1.41o, National Institutes of Health, USA).

### Statistical analysis

Four independent experiments were performed for rtPCR, and for each experiment, three PCR runs, each in triplicate, were analyzed. For the Sudan staining three independent experiments were performed, each analyzing 10 wells for each condition and for each 10.000 internodes were counted. Statistical myelin quantification comprises three independent experiments, internodal length is displayed as one representative experiment; an analysis of variance between the three independent experiments revealed no significant difference. For immunocytochemistry (IL-17R, MHCI, TAPII, MHCII) three independent experiments were performed, each with 12 wells for each condition. The graphs depict one representative experiment with the analysis of 50 sectors of the cell layer in each well. An analysis of variance between the three independent experiments revealed no significant difference.

Immunocytochemistry of neurofilament is graphed for three independent experiments.

Electron Microscopy was performed in at least three independent experiments; graph for viability analysis shows results of three independent experiments in a 96-well plate (24 samples per condition). Gelatinase zymography graphs show densitometry of one representative experiment out of four; due to the difficulties in comparing different zymography-runs (for example, background), we present the results of one experiment.

**Figure 1:**
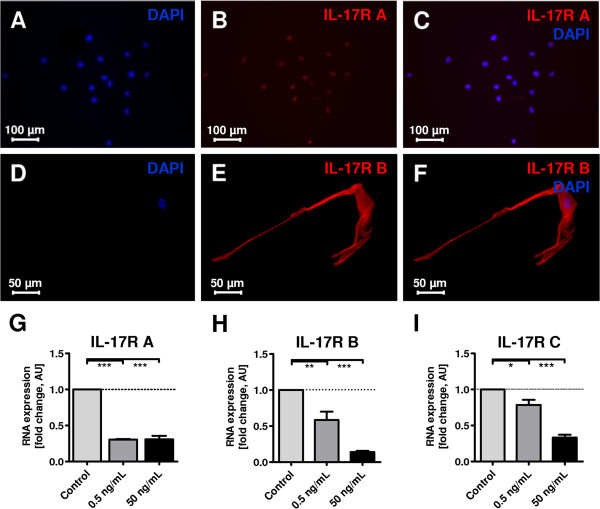
**Detection of interleukin 17 (IL-17) receptors (R) IL-17R A and IL-17R B on mouse Schwann cells (SCs), using immunocytochemistry and rtPCR analysis. (A-C)** immunocytochemistry for IL-17R A, IL-17R B with mainly membrane-bound expression **(D-F)**. **(G-I)** Real-time PCR results of IL-17R mRNA expression of mouse dorsal root ganglia (DRG) co-cultures. Cultures were treated with IL-17 for 21 days at the indicated concentrations. Stimulation gave rise to a strong decrease in IL-17R A expression at the mRNA level under stimulation with 0.5 and 50 mg/mL IL-17, with a remaining expression of 30% (***P ≤0.001). A dose-dependent decrease was detected for IL-17R B and IL-17R C. The remaining expression of IL-17R B was 58% with 0.5 ng/mL IL-17 (**P ≤0.01) and 14% with 50 ng/mL IL-17 (***P ≤0.001). Similarly, the expression of IL-17R C was significantly reduced at the mRNA level to 78% for 0.5 ng/mL IL-17 (*P ≤0.05) and 33% for 50 ng/mL IL-17 (***P ≤0.001). For all three receptors and the concentrations applied, an analysis of variance between the three independent experiments revealed no significant difference. AU, arbitrary unit; DAPI, diamidino-2-phenylindole.

**Figure 2:**
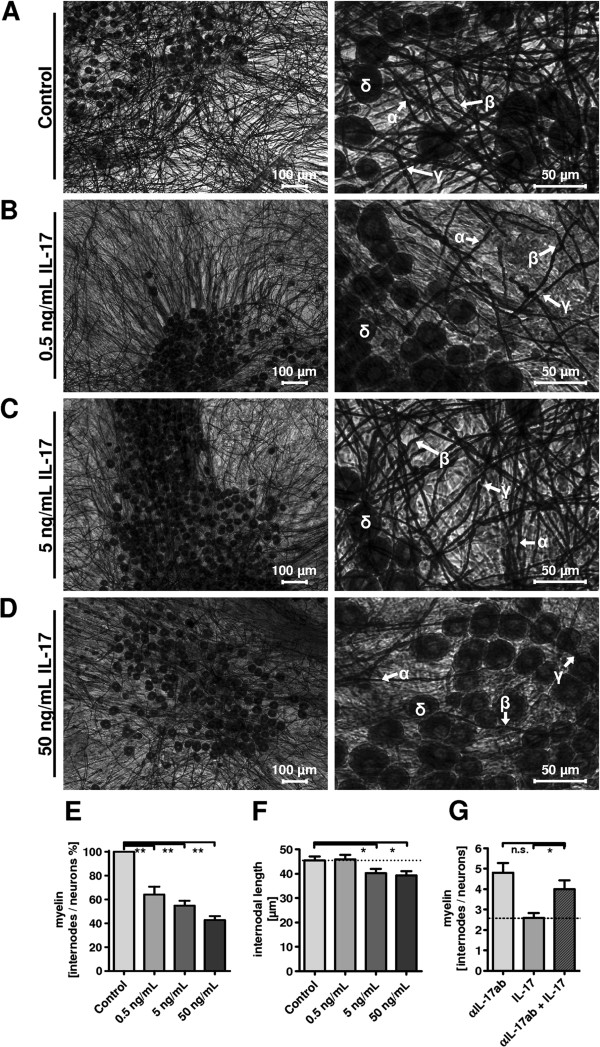
**Myelin morphology and quantification after interleukin 17 (IL-17) treatment. (A-E)** For myelin quantification, mouse dorsal root ganglia (DRG) co-cultures were exposed to IL-17 for 21 days at the indicated concentrations and subsequently stained, using Sudan black dye. Images show myelin layer (α) and Schwann cell body (β) are separated from the next internode by Ranvier’s node (γ) and neuronal cell bodies (δ). In analysis of three independent experiments, quantification revealed a myelin ratio (internodes to neurons) reduction to 66% for 0.5 ng/mL IL-17 (**P = <0.01), to 55% for 5 ng/mL IL-17 (**P = <0.01), and to 42% for 50 ng/mL IL-17 (**P = <0.01), each compared with control stimulations. **(F)** Internodal distances after exposure to IL-17 revealed a significant decrease with 5 ng/mL IL-17 (*P = <0.05) and 50 ng/mL IL-17 (*P = <0.05). Analysis of variances between the independent experiments revealed no significant difference. **(G)** Myelin quantification of Sudan-stained DRG co-cultures after treatment with IL-17, an IL-17-neutralizing antibody (αIL-17ab), and co-stimulation of three independent experiments, respectively. The myelin-inhibitory effect of IL-17 was reduced by 81% after supplementation with αIL-17ab. Myelination was restored to the base level, similar to treatment with the antibody alone.

**Figure 3:**
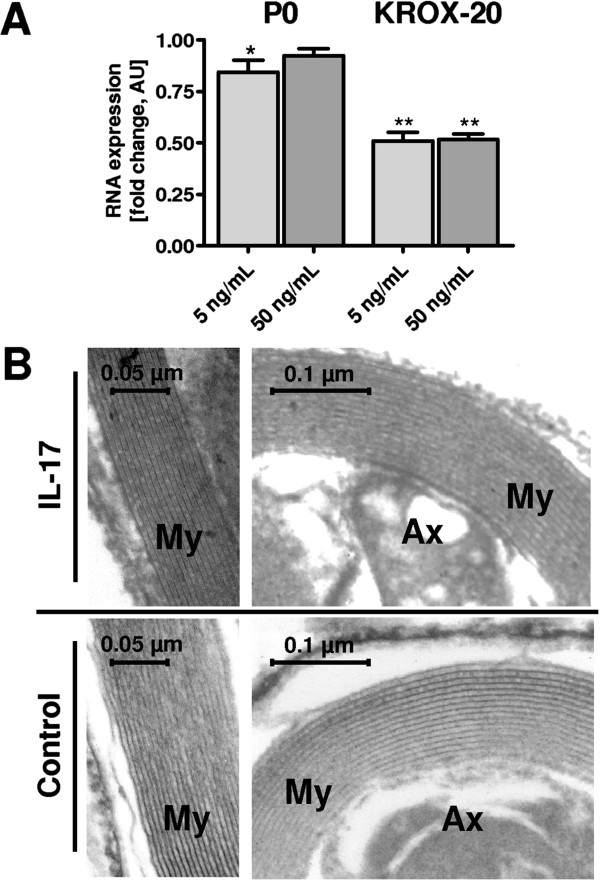
**mRNA expression of myelin genes P0, and KROX-20 of rat Schwann cells (rSCs) and myelin morphology after stimulation with interleukin 17 (IL-17). (A)** mRNA expression of myelin genes P0 and KROX-20 is normalized to glyceraldehyde 3-phosphate dehydrogenase (GAPDH) and to control cultures. A significant decrease in KROX-20 mRNA expression to 51% (**P ≤0.01) was seen on stimulation with 5 and 50 ng/mL IL-17. P0 mRNA showed a significant down-regulation to 84% expression with 5 ng/mL IL-17 (*P ≤0.05) and a non-significant down-regulation to 92% expression with 50 ng/mL IL-17. For P0 and KROX-20 and both concentrations applied; an analysis of variance between the three independent experiments revealed no significant difference. **(B)** Ultrastructure of the myelin layer of dorsal root ganglia (DRG) co-cultures after IL-17 stimulation (5 ng/mL IL-17, top row) and control cultures (bottom row). Cultures were stimulated from day 6 after preparation and fixed after 28 days *in vitro*. The left images depict a longitudinal section and the right images a traverse section referring to the axonal fiber. Myelin showed no morphological alterations after IL-17 stimulation. AU, arbitrary unit; Ax, axon; My, myelin layer.

**Figure 4:**
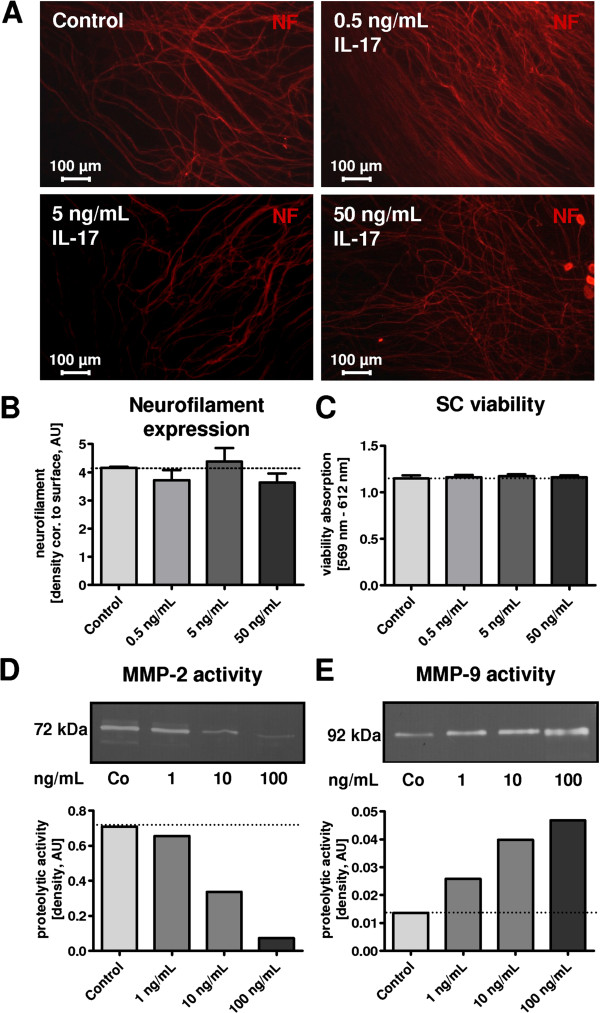
**Neuronal outgrowth, Schwann cell (SC) viability, and Matrix metalloproteinases (MMP) activity in Interleukin 17 (IL-17) treated cultures. (A)** Neuronal outgrowth was assessed in mouse dorsal root ganglia (DRG) co-cultures after exposure for 21 days to IL-17 at the indicated concentrations. Representative neurofilament (NF)-stained images of the cultures are depicted. **(B)** Quantification of NF, using densitometry analysis, normalized to background fluorescence and control stimulation. Analysis did not reveal a significant alteration in neuronal outgrowth. **(C)** SC viability was analyzed after IL-17 stimulation for 7 days at the indicated concentrations. Concentrations between 0.5 and 50 ng/mL did not significantly alter SC viability. Graph for viability and neurofilament analysis shows results of three independent experiments **(D, E)**. MMP activity in IL-17-treated DRG co-culture supernatants was analyzed using gelatine zymography and quantified densitometrically. Representative zymography with the densitometry analysis is depicted. Overall, three independent experiments were performed and analyzed, exhibiting comparable results. AU, arbitrary unit. IL-17 led to a dose-dependent increase in inflammatory MMP-9 activity (**E**; 92 kDa), whereas the active and the inactive form of MMP-2 (**D**; MMP-2 inactive: 72 kDa, active: 62 kDa) decreased in activity.

**Figure 5:**
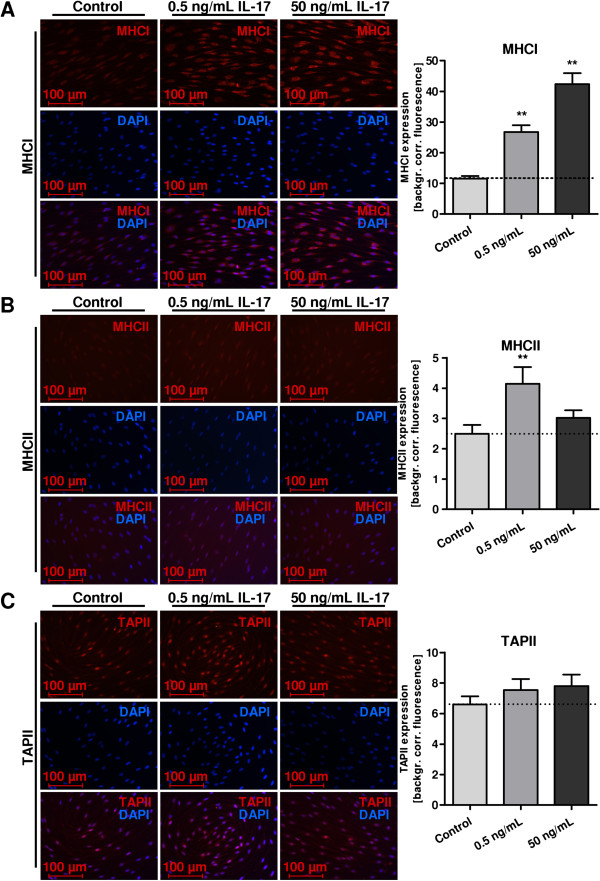
**Major histocompatibility complex (MHC) I and II as well as Transporter associated with antigen presentation II (TAPII) were analyzed, using immunocytochemistry on rat Schwann cells (SCs).** Corresponding merges are shown in the bottom rows. Treatment of SCs with IL-17 was performed at concentrations of 0.5 and 50 ng/mL. Graphs to the right show densitometry quantification. SCs showed expression of MHCI > TAPII > MHCII, which increased after IL-17 treatment. **(A)** MHCI was mainly detected in the cytoplasm and the expression increased in a dose-dependent manner after IL-17 treatment, significant for 0.5 ng/mL and 50 ng/mL (**P ≤0.01). **(B)** MHCII revealed a fainter basic expression emphasizing the nucleus and was found significantly increased after 0.5 ng/mL IL-17 stimulation (**P ≤ 0.01). **(C)** TAPII was detected in the nucleus and cytoplasm. We detected a dose-dependent tendency but no significantly increased expression after IL-17 stimulation. For MHCI, TAPII and MHCII and the concentrations applied, analysis of variance between the independent experiments revealed no significant difference. DAPI, 4′, 6-diamidino-2-phenylindole.

Statistical analysis (mean, standard deviation, and *P* values) was performed with GraphPad Prism software version 4.0 (GraphPad Software, Inc., La Jolla, CA, USA). Calculations involved unpaired t-tests respectively at 95% confidence interval, one-way ANOVA with Bonferroni and Dunnett’s test (Figure 1G-I, Figure 2E-G, Figure 3A, Figure 4B-C, Figure 5A-C), with *P* <0.05 considered as statistically significant (*P* values: * <0.05, ** <0.01, *** <0.001).

## Results

### Schwann cells express IL-17 receptors

To evaluate the molecular target of IL-17, IL-17R A and IL-17R B were analyzed on SCs using immunocytochemistry. No inflammatory stimulus was required to induce IL-17R expression on mouse SCs (mSCs; passage nine). mSCs revealed that constitutive expression of IL-17R A, IL-17R B was mainly detectable in a membrane-bound manner (Figure 1A-F). To evaluate functional expression, stimulation of mouse DRG cultures with IL-17 for 21 days was performed (Figure 1G-I). We used rSCs for the rtPCR analysis due to the fact that primary cultures from rSCs are standardized and provide more reproducible results (Stettner *et al*. [29]). Simulation gives rise to a strong decrease in IL-17R A expression on mRNA levels after stimulation with 0.5 and 50 ng/mL IL-17 (Figure 1G). Expression levels of both IL-17R B (Figure 1H) and IL-17 receptor C (IL-17R C) (Figure 1I) decreased in a dose-dependent manner. These results suggest a functional expression of the receptor.

### IL-17 inhibits myelination *in vitro*

Mouse DRG co-cultures were treated with IL-17 for 21 days followed by Sudan black staining (Figure 2A-D) to assess the impact on myelination. The ratio of internodes to neurons decreased to 66%, 55%, and 42% with 0.5, 5 and 50 ng/mL IL-17, respectively (Figure 2E). Accordingly, the internodal distance, a parameter for quantitative myelin synthesis, was reduced significantly following stimulation with 5 and 50 ng/mL IL-17 (Figure 2F). Measurement of fiber diameter in Sudan stained cultures did not reveal a significant difference for the IL-17-treated cultures (data not shown). Co-stimulation of DRG co-cultures with IL-17 and an IL-17-neutralizing antibody reduced the myelin inhibitory effect by about 81% (Figure 2G).

mRNA expression of genes associated with myelination of rSCs was analyzed after stimulation with IL-17 for 10 days. The mRNA expression of KROX-20 decreased significantly with 5 and 50 ng/mL IL-17 (Figure 3A). For P0, the mRNA expression decreased significantly with 5 ng/mL IL-17 and decreased non-significantly with 50 ng/mL IL-17 (Figure 3A). Evaluation of myelin morphology using electron microscopy did not reveal any morphological alterations when compared with control cultures (Figure 3B).

### IL-17 does not impede neuronal outgrowth and cell survival

To exclude a direct effect of IL-17 on neuronal outgrowth and a consecutive effect on myelin synthesis, we performed neurofilament (NF) staining and quantification in mouse DRG co-cultures after exposure to IL-17 for 21 days. Neuronal fibers did not show a significant reduction in NF after IL-17 exposure using densitometry analysis, normalized to background fluorescence (Figure 4A, B).

To further exclude a direct effect of IL-17 on SC survival, viability assays were performed in rSCs. Stimulation with 0.5 to 50 ng/mL IL-17 did not cause any significant alterations in SC viability (Figure 4C).

To analyze additional mediators of inflammation and myelination, the activity of MMPs was assessed. IL-17-treated DRG co-culture supernatants revealed a significant dose-dependent downregulation of MMP-2 activity and a dose-dependent upregulation of MMP-9 activity, assessed in gelatine zymography and quantified using densitometry (Figure 4D, E).

### IL-17 increases major histocompatibility complex and transporter associated with antigen presentation expression

To assess the potential of IL-17 to modify the expression of molecules associated with antigen transformation and presentation, MHCI and MHCII, as well as TAPII, were analyzed using immunocytochemistry and quantified using densitometry. SCs displayed expression of all three molecules: MHCI > TAPII > MHCII. MHCI expression was significantly increased in a dose-dependent manner after treatment with IL-17 for 72 h (Figure 5A). Compared to MHCI, MHCII exhibited a fainter expression under control conditions; its expression was slightly but not significantly increased after 50 ng/mL IL-17, but revealed a significant increase at a concentration of 0.5 ng/mL IL-17 (Figure 5B). The expression of TAPII was slightly, though not significantly, increased in a dose-dependent manner after stimulation with IL-17 (Figure 5C).

## Discussion

IL-17 is present in demyelinating peripheral nerve inflammation as well as in demyelinating chronic pain conditions [2,3,8,9]. IL-17 has also been associated with GBS, a mainly acute demyelination condition of the PNS [1,12,13,34]. Several studies have revealed that IL-17 is present primarily during the high-demyelination stage of these inflammatory and autoimmune diseases [8-10,12,35], suggesting that a direct SC-driven demyelinating effect of IL-17 is possible. A prerequisite for a direct response of SCs to IL-17 is the expression of IL-17R on SCs, which has been confirmed at both the RNA and protein level.

Stimulation with IL-17 led to a downregulation on mRNA level of all IL-17R analyzed. Despite the growing knowledge about the relevance of IL-17 in various diseases and the fact that the IL-17 receptor is ubiquitously expressed, little is known about the IL-17R regulation. Presumably IL-17 signaling is strictly controlled in order to prevent inflammatory disorders [36,37]. It is known that IL-21 preferentially induces IL-17R, compared with IL-2. A blockade of the PI3K pathway led to the upregulation of IL-17R A and constitutive Akt activation is associated with suppressed IL-17RA expression [38]. Whether the decrease in expression after IL-17 stimulation detected in the current study is due to a feedback loop, which is known for other receptors [39], self-limiting the signal, is speculative but the data suggests a functional expression of the receptors on SCs.

In this study, we analyzed the influence of IL-17 on SC myelination using the DRG co-culture model. Stimulation with IL-17 resulted in reduced myelin synthesis as well as a decrease in the length of internodal segments, an effect that was eliminated by the addition of an IL-17-neutralizing antibody.

Several inflammatory mediators are able to induce SC de-differentiation or activation *in vivo* and *in vitro* as a response to pathogens or after mechanical nerve damage [40,41]. In addition to reduced myelination under IL-17 influence, we also detected reduced expression of the myelinating and pro-myelinating key genes P0 and KROX-20, for P0 in a concentration of 5 ng/mL and for KROX-20 in both concentrations applied (5 ng/mL and 50 ng/mL). P0, or myelin protein zero (MPZ), is a structural component of the myelin layer, necessary for myelin compaction, whereas KROX-20 is a myelin-associated transcription factor for pro-myelinating pathways [41-47]. Krox-20 is activated in SCs before the onset of myelination and its disruption blocks SCs at an early stage in their differentiation, while radial sorting and attainment of 1:1 relationships between axons and SC are mainly unaffected [15,45,48,49]. KROX-20 activates a large number of myelin genes and suppresses molecules of the immature SC-stage. KROX-20 is a major candidate for holding the balance between two opposing transcriptional programs - the immature phenotype versus the myelinating SC [41]. The discrepancy between the KROX-20 and the P0 expression is astonishing but may be due to a P0 independent signaling pathway or just due to the fact that the 5 ng/mL IL-17 concentration is closer the local IL-17 concentration in the inflamed nerve. Robust data on the local IL-17 concentration within the inflamed peripheral nerve do not exist so far.

Various compounds are known to mediate direct myelin degrading properties. IL-17 triggers the expression of inducible nitric oxide (NO) synthase, leading to cytotoxicity [50,51]. In epithelial, endothelial, and fibroblastic cells IL-17 induces the secretion of cytokines, such as IL-6, IL-8, and granulocyte colony-stimulating factor, as well as prostaglandin E2 (PGE2) [52]. IL-17 also induces COX-2-dependent PGE2 synthesis and osteoclast differentiation factor gene expression, which leads to bone resorption in rheumatoid arthritis patients [53]. IL-17 also contributes to the disruption of the blood- brain barrier by augmented production of reactive oxygen species mediated by nicotinamide adenine dinucleotide phosphate oxidase and xanthine oxidases. The resulting oxidative stress finally leads to interaction with the cytoskeleton [54].

Thus, a direct toxic effect of IL-17 on the myelin layer is feasible. To determine if oxidative stress was underlying the reduced myelin synthesis following IL-17 treatment, we analyzed the structure of myelin cultures to detect myelin debris, an observation that would support the thesis of NO-induced myelin destruction. We detected a reduced amount of myelin, but did not observe any morphological changes in myelin layers after IL-17 treatment. Although our current findings do not provide evidence of myelin destruction, we cannot exclude a direct component of IL-17 induced myelin destruction. Still, usually infiltrating macrophages cause myelin debris and scavenge at the site of inflammation [24]; and this cellular part of the immune system is not present in the DRG/SC co-culture system that was used in our study - this gives cause for another explanation of the scenario.

To further exclude an indirect effect of reduced neuronal contact, which promotes myelination, we analyzed neuronal outgrowth but found no significant changes, therefore making this thesis unlikely. We also cannot completely rule out that IL-17 simply leads to a delay in myelin synthesis, but on the basis of the results presented this seems to be unlikely.

It has been suggested that the increase in IL-17^+^ cells following sciatic nerve and spinal cord injury induces SC apoptosis [55]. In contrast to these results, we found that SC viability was not affected after IL-17 stimulation, suggesting an apoptosis-independent mechanism for the decrease in myelination that we observed.

Furthermore, we detected a decreased MMP-2 activity in DRG culture supernatants, which has been shown relevant for the induction of myelin synthesis [30], while pro-inflammatory MMP-9 activity was increased. Our results are in line with studies reporting an induction of MMP-9 by IL-17 in various organ systems [56,57].

Since SCs possess the molecular machinery for antigen transformation and presentation, we further analyzed the expression of molecules associated with these properties. MHCI was detected mainly in the cytoplasm, and the expression was increased by IL-17 in a dose-dependent manner. Compared with MHCI, MHCII revealed a fainter basic expression and also an increase in protein expression after IL-17, with a significantly increased expression at 0.5 ng/mL IL-17, whereas 50 ng/mL just revealed a tendency without statistical significance. Antigenic peptides are transferred to the endoplasmic reticulum by TAP. We found that the expression of TAPII increased slightly in a dose-dependent manner following IL-17 treatment, but without statistical significance in densitometric analysis.

Thus, we assume that the capacity of SCs to activate CD8^+^ and CD4^+^ T cells, and therefore to act as antigen-presenting cells in a condition of inflammation, was increased. In accord with our findings, SCs express major histocompatibility complex molecules and increase the expression of these molecules under pro-inflammatory conditions *in vivo* and *in vitro*[17,18,58-70]. Whether the change of MHCII and TAP expression in SC after IL-17 stimulation is functional is notional. The results warrant further functional analysis of MHCII expression in SCs.

Corroborating our results, regulated expression of the intracellular antigen-processing machinery in peripheral nerve sections from GBS patients was recently shown (Meyer zu Horste *et al*. [17]), and SCs most likely act as non-professional antigen-presenting cells under certain conditions. The IL-17-induced increased expression can be interpreted as an immunological alignment of SCs. This modulation of SC homeostasis by inflammatory mediators was reported for interleukins, iNOS, COX-2, and MMPs as a response to the surrounding environment [20-24].

IL-17 led to no reduction of SC viability, showed no effect on NF expression, and did not change the structural myelin formation, but significantly interfered with SC-mediated myelination. Negative regulators of myelination which do not necessarily initiate apoptosis such as c-Jun, Notch, Sox-2, Pax-3, Id2, Krox-24, and Egr-3 are known. They are found downregulated after initiation of myelination and may be reactivated after PNS injury causing SC dedifferentiation [41]. Further inflammatory mediators, such as TNF-α, can promote phenotype reversion of mature to immature SCs [25-27,41].

It was suggested before that negative regulators of SC-myelination may foster neuronal survival and axonal re-growth but actively suppress myelination. In the context of neuropathies, such pathways may cause further harm [41].

## Conclusions

In summary, our findings demonstrate the ability of IL-17 to reduce SC-mediated myelination, an effect that does not appear to involve an indirect effect caused by either a reduced neuronal stimulus for myelination or a toxic effect, but rather a direct reprogramming of SC differentiation. This latter effect may contribute to Wallerian degeneration and equipping of the inflammatory facilities of SCs in order to modulate the process of inflammation.

These data provide new insights into the role of IL-17 in the inflammatory response in the PNS that could be useful in the development of targeted therapies.

## Abbreviations

AU: arbitrary unit; AX: axon; CIDP: chronic inflammatory demyelinating polyneuropathy; CSF: cerebrospinal fluid; DAPI: diamidino-2-phenylindole; DMEM: Dulbecco’s modified eagle’s medium; DRG: dorsal root ganglia; FCS: fetal calf serum; FKS: forskolin; GAPDP: glyceraldehyde 3-phosphate dehydrogenase; GBS: Guillain-Barré syndrome; Glut: L-glutamine; HBSS: Hank’s balanced salt solution; HEPES: 4-(2-hydroxyethyl)-1-piperazine ethane sulfonic acid; HS: horse serum; iNOS: inducible nitric oxide synthase; MEM: minimal essential media; MHC: major histocompatibility complex; MMP: matrix metalloproteinase; mSC: mouse Schwann cell; MY: myelin layer; NF: neurofilament; NF-L: neurofilament L; NGS: natural goat serum; PBMCs: peripheral blood mononuclear cells; PBS: phosphate-buffered saline solution; PEB: pituitary extract bovine; PFA: paraformaldehyde; PNS: peripheral nervous system; P/S: penicillin/streptomycin; rSCs: rat Schwann cells; RT: room temperature; rtPCR: real-time polymerase chain reaction; SC: Schwann cell; SDS/PAGE: sodium dodecyl sulfate-polyacrylamide gel electrophoresis; TAP: transporter associated with antigen presentation; TLRs: toll-like receptors; TNF-α: tumor necrosis factor α.

## Competing interests

The authors declare that they have no competing interests.

## Authors’ contributions

MS and BK were responsible for the study concept and design; MS, BL, KW, HH, and JW were responsible for the analysis and interpretation of data; MS and BK drafted the manuscript; MS, KW, TD, AM, HH, and BK critically revised the manuscript for important intellectual content; MS, BL, KW, JW, and TD supported administratively and technically; MS, and BK supervised the study. All authors have read and approved the final version of the manuscript.

## References

[B1] Lu MO Zhu J The role of cytokines in guillain-barre syndrome J Neurol 2011 258 533 548 10.1007/s00415-010-5836-5 21104265

[B2] Kleinschnitz C Hofstetter HH Meuth SG Braeuninger S Sommer C Stoll G T cell infiltration after chronic constriction injury of mouse sciatic nerve is associated with interleukin-17 expression Exp Neurol 2006 200 480 485 10.1016/j.expneurol.2006.03.014 16674943

[B3] Kim CF Moalem-Taylor G Interleukin-17 contributes to neuroinflammation and neuropathic pain following peripheral nerve injury in mice J Pain 2011 12 370 383 20889388 10.1016/j.jpain.2010.08.003

[B4] Ferretti S Bonneau O Dubois GR Jones CE Trifilieff A IL-17, produced by lymphocytes and neutrophils, is necessary for lipopolysaccharide-induced airway neutrophilia: IL-15 as a possible trigger J Immunol 2003 170 2106 2112 12574382 10.4049/jimmunol.170.4.2106

[B5] Molet S Hamid Q Davoine F Nutku E Taha R Page N Olivenstein R Elias J Chakir J IL-17 is increased in asthmatic airways and induces human bronchial fibroblasts to produce cytokines J Allergy Clin Immunol 2001 108 430 438 10.1067/mai.2001.117929 11544464

[B6] Weaver CT Hatton RD Mangan PR Harrington LE IL-17 family cytokines and the expanding diversity of effector T cell lineages Annu Rev Immunol 2007 25 821 852 10.1146/annurev.immunol.25.022106.141557 17201677

[B7] Tzartos JS Friese MA Craner MJ Palace J Newcombe J Esiri MM Fugger L Interleukin-17 production in central nervous system-infiltrating T cells and glial cells is associated with active disease in multiple sclerosis Am J Pathol 2008 172 146 155 10.2353/ajpath.2008.070690 18156204 PMC2189615

[B8] Madia F Frisullo G Nociti V Conte A Luigetti M Del Grande A Patanella AK Iorio R Tonali PA Batocchi AP Sabatelli M pSTAT1, pSTAT3, and T-bet as markers of disease activity in chronic inflammatory demyelinating polyradiculoneuropathy J Peripher Nerv Syst 2009 14 107 117 10.1111/j.1529-8027.2009.00220.x 19691533

[B9] Mei FJ Ishizu T Murai H Osoegawa M Minohara M Zhang KN Kira J Th1 shift in CIDP versus Th2 shift in vasculitic neuropathy in CSF J Neurol Sci 2005 228 75 85 10.1016/j.jns.2004.10.001 15607214

[B10] Chi LJ Xu WH Zhang ZW Huang HT Zhang LM Zhou J Distribution of Th17 cells and Th1 cells in peripheral blood and cerebrospinal fluid in chronic inflammatory demyelinating polyradiculoneuropathy J Peripher Nerv Syst 2010 15 345 356 10.1111/j.1529-8027.2010.00294.x 21199106

[B11] Zhang Z Zhang ZY Schluesener HJ Compound A, a plant origin ligand of glucocorticoid receptors, increases regulatory T cells and M2 macrophages to attenuate experimental autoimmune neuritis with reduced side effects J Immunol 2009 183 3081 3091 10.4049/jimmunol.0901088 19675162

[B12] Gries M Davies L Liu Y Bachhuber A Spiegel J Dillmann U Hartmann T Fassbender K Walter S Response of Toll-like receptors in experimental Guillain-Barre syndrome: a kinetic analysis Neurosci Lett 2012 518 154 160 10.1016/j.neulet.2012.04.077 22579825

[B13] Li S Yu M Li H Zhang H Jiang Y IL-17 and IL-22 in cerebrospinal fluid and plasma are elevated in Guillain-Barre syndrome Mediators Inflamm 2012 2012 260473 23091305 10.1155/2012/260473 PMC3468147

[B14] Fossiez F Banchereau J Murray R Van Kooten C Garrone P Lebecque S Interleukin-17 Int Rev Immunol 1998 16 541 551 10.3109/08830189809043008 9646176

[B15] Jessen KR Mirsky R Salzer J Introduction. Schwann cell biology Glia 2008 56 1479 1480 10.1002/glia.20779 18803316

[B16] Barcia C Sr Mitxitorena I Carrillo-de Sauvage MA Gallego JM Perez-Valles A Barcia CJ Barcia C Sr Mitxitorena I Carrillo-de Sauvage MA Gallego JM Perez-Valles A Barcia C Jr Imaging the microanatomy of astrocyte-T-cell interactions in immune-mediated inflammation Front Cell Neurosci 2013 7 58 23641198 10.3389/fncel.2013.00058 PMC3639405

[B17] Meyer zu Horste G Heidenreich H Lehmann HC Ferrone S Hartung HP Wiendl H Kieseier BC Expression of antigen processing and presenting molecules by Schwann cells in inflammatory neuropathies Glia 2010 58 80 92 10.1002/glia.20903 19544394 PMC3480724

[B18] Meyer zu Horste G Heidenreich H Mausberg AK Lehmann HC ten Asbroek AL Saavedra JT Baas F Hartung HP Wiendl H Kieseier BC Mouse Schwann cells activate MHC class I and II restricted T-cell responses, but require external peptide processing for MHC class II presentation Neurobiol Dis 2010 37 483 490 10.1016/j.nbd.2009.11.006 19914379

[B19] Goethals S Ydens E Timmerman V Janssens S Toll-like receptor expression in the peripheral nerve Glia 2010 58 1701 1709 10.1002/glia.21041 20578041

[B20] Bolin LM Verity AN Silver JE Shooter EM Abrams JS Interleukin-6 production by Schwann cells and induction in sciatic nerve injury J Neurochem 1995 64 850 858 7830079 10.1046/j.1471-4159.1995.64020850.x

[B21] Chattopadhyay S Myers RR Janes J Shubayev V Cytokine regulation of MMP-9 in peripheral glia: implications for pathological processes and pain in injured nerve Brain Behav Immun 2007 21 561 568 10.1016/j.bbi.2006.10.015 17189680 PMC2865892

[B22] Levy D Hoke A Zochodne DW Local expression of inducible nitric oxide synthase in an animal model of neuropathic pain Neurosci Lett 1999 260 207 209 10.1016/S0304-3940(98)00982-3 10076904

[B23] Takahashi M Kawaguchi M Shimada K Konishi N Furuya H Nakashima T Cyclooxygenase-2 expression in Schwann cells and macrophages in the sciatic nerve after single spinal nerve injury in rats Neurosci Lett 2004 363 203 206 10.1016/j.neulet.2004.03.040 15182944

[B24] Tofaris GK Patterson PH Jessen KR Mirsky R Denervated Schwann cells attract macrophages by secretion of leukemia inhibitory factor (LIF) and monocyte chemoattractant protein-1 in a process regulated by interleukin-6 and LIF J Neurosci 2002 22 6696 6703 12151548 10.1523/JNEUROSCI.22-15-06696.2002 PMC6758146

[B25] Boyle K Azari MF Cheema SS Petratos S TNFalpha mediates Schwann cell death by upregulating p75NTR expression without sustained activation of NFkappaB Neurobiol Dis 2005 20 412 427 10.1016/j.nbd.2005.03.022 15905096

[B26] Pollheimer J Knofler M Signalling pathways regulating the invasive differentiation of human trophoblasts: a review Placenta 2005 26 Suppl A S21 S30 15837062 10.1016/j.placenta.2004.11.013

[B27] Schafers M Geis C Brors D Yaksh TL Sommer C Anterograde transport of tumor necrosis factor-alpha in the intact and injured rat sciatic nerve J Neurosci 2002 22 536 545 11784800 10.1523/JNEUROSCI.22-02-00536.2002 PMC6758659

[B28] Brockes JP Fields KL Raff MC Studies on cultured rat Schwann cells. I. Establishment of purified populations from cultures of peripheral nerve Brain Res 1979 165 105 118 10.1016/0006-8993(79)90048-9 371755

[B29] Stettner M Wolffram K Mausberg AK Wolf C Heikaus S Derksen A Dehmel T Kieseier BC A reliable in vitro model for studying peripheral nerve myelination in mouse J Neurosci Methods 2013 214 69 79 10.1016/j.jneumeth.2013.01.009 23348045

[B30] Lehmann HC Kohne A Bernal F Jangouk P Meyer Zu Horste G Dehmel T Hartung HP Previtali SC Kieseier BC Matrix metalloproteinase-2 is involved in myelination of dorsal root ganglia neurons Glia 2009 57 479 489 10.1002/glia.20774 18814268

[B31] Dehmel T Janke A Hartung HP Goebel HH Wiendl H Kieseier BC The cell-specific expression of metalloproteinase-disintegrins (ADAMs) in inflammatory myopathies Neurobiol Dis 2007 25 665 674 10.1016/j.nbd.2006.11.008 17207628

[B32] Stettner M Dehmel T Mausberg AK Kohne A Rose CR Kieseier BC Levetiracetam exhibits protective properties on rat Schwann cells in vitro J Peripher Nerv Syst 2011 16 250 260 10.1111/j.1529-8027.2011.00355.x 22003940

[B33] Kieseier BC Kiefer R Clements JM Miller K Wells GM Schweitzer T Gearing AJ Hartung HP Matrix metalloproteinase-9 and −7 are regulated in experimental autoimmune encephalomyelitis Brain 1998 121 Pt 1 159 166 9549496 10.1093/brain/121.1.159

[B34] Liang SL Wang WZ Huang S Wang XK Zhang S Wu Y Th17 helper cell and T-cell immunoglobulin and mucin domain 3 involvement in Guillain-Barre syndrome Immunopharmacol Immunotoxicol 2012 34 1039 1046 10.3109/08923973.2012.697469 22738814

[B35] Kim HJ Jung CG Jensen MA Dukala D Soliven B Targeting of myelin protein zero in a spontaneous autoimmune polyneuropathy J Immunol 2008 181 8753 8760 19050296 10.4049/jimmunol.181.12.8753 PMC2745060

[B36] Basu R Hatton RD Weaver CT The Th17 family: flexibility follows function Immunol Rev 2013 252 89 103 10.1111/imr.12035 23405897 PMC3607325

[B37] Song X Qian Y The activation and regulation of IL-17 receptor mediated signaling Cytokine 2013 62 175 182 10.1016/j.cyto.2013.03.014 23557798

[B38] Lindemann MJ Hu Z Benczik M Liu KD Gaffen SL Differential regulation of the IL-17 receptor by gammac cytokines: inhibitory signaling by the phosphatidylinositol 3-kinase pathway J Biol Chem 2008 283 14100 14108 10.1074/jbc.M801357200 18348982 PMC2376247

[B39] Alimirah F Chen J Xin H Choubey D Androgen receptor auto-regulates its expression by a negative feedback loop through upregulation of IFI16 protein FEBS Lett 2006 580 1659 1664 10.1016/j.febslet.2006.02.015 16494870

[B40] Stoll G Jander S Myers RR Degeneration and regeneration of the peripheral nervous system: from Augustus Waller’s observations to neuroinflammation J Peripher Nerv Syst 2002 7 13 27 10.1046/j.1529-8027.2002.02002.x 11939348

[B41] Jessen KR Mirsky R Negative regulation of myelination: relevance for development, injury, and demyelinating disease Glia 2008 56 1552 1565 10.1002/glia.20761 18803323

[B42] Hossain S de la Cruz-Morcillo MA Sanchez-Prieto R Almazan G Mitogen-activated protein kinase p38 regulates Krox-20 to direct Schwann cell differentiation and peripheral myelination Glia 2012 60 1130 1144 10.1002/glia.22340 22511272

[B43] Kipanyula MJ Woodhoo A Rahman M Payne D Jessen KR Mirsky R Calcineurin-nuclear factor of activated T cells regulation of Krox-20 expression in Schwann cells requires elevation of intracellular cyclic AMP J Neurosci Res 2013 91 105 115 23073893 10.1002/jnr.23131 PMC5722200

[B44] Salzer JL Switching myelination on and off J Cell Biol 2008 181 575 577 10.1083/jcb.200804136 18490509 PMC2386097

[B45] Jessen KR Mirsky R The origin and development of glial cells in peripheral nerves Nat Rev Neurosci 2005 6 671 682 10.1038/nrn1746 16136171

[B46] Lee M Brennan A Blanchard A Zoidl G Dong Z Tabernero A Zoidl C Dent MA Jessen KR Mirsky R P0 is constitutively expressed in the rat neural crest and embryonic nerves and is negatively and positively regulated by axons to generate non-myelin-forming and myelin-forming Schwann cells, respectively Mol Cell Neurosci 1997 8 336 350 10.1006/mcne.1996.0589 9073396

[B47] Stewart HJ Bradke F Tabernero A Morrell D Jessen KR Mirsky R Regulation of rat Schwann cell Po expression and DNA synthesis by insulin-like growth factors in vitro Eur J Neurosci 1996 8 553 564 10.1111/j.1460-9568.1996.tb01240.x 8963447

[B48] Jessen KR Mirsky R Signals that determine Schwann cell identity J Anat 2002 200 367 376 10.1046/j.1469-7580.2002.00046.x 12090403 PMC1570691

[B49] Topilko P Schneider-Maunoury S Levi G Baron-Van Evercooren A Chennoufi AB Seitanidou T Babinet C Charnay P Krox-20 controls myelination in the peripheral nervous system Nature 1994 371 796 799 10.1038/371796a0 7935840

[B50] Miljkovic D Trajkovic V Inducible nitric oxide synthase activation by interleukin-17 Cytokine Growth Factor Rev 2004 15 21 32 10.1016/j.cytogfr.2003.10.003 14746811

[B51] Trajkovic V Stosic-Grujicic S Samardzic T Markovic M Miljkovic D Ramic Z Mostarica Stojkovic M Interleukin-17 stimulates inducible nitric oxide synthase activation in rodent astrocytes J Neuroimmunol 2001 119 183 191 10.1016/S0165-5728(01)00391-5 11585620

[B52] Fossiez F Djossou O Chomarat P Flores-Romo L Ait-Yahia S Maat C Pin JJ Garrone P Garcia E Saeland S Blanchard D Gaillard C Das Mahapatra B Rouvier E Golstein P Banchereau J Lebecque S T cell interleukin-17 induces stromal cells to produce proinflammatory and hematopoietic cytokines J Exp Med 1996 183 2593 2603 10.1084/jem.183.6.2593 8676080 PMC2192621

[B53] Kotake S Udagawa N Takahashi N Matsuzaki K Itoh K Ishiyama S Saito S Inoue K Kamatani N Gillespie MT Martin TJ Suda T IL-17 in synovial fluids from patients with rheumatoid arthritis is a potent stimulator of osteoclastogenesis J Clin Invest 1999 103 1345 1352 10.1172/JCI5703 10225978 PMC408356

[B54] Huppert J Closhen D Croxford A White R Kulig P Pietrowski E Bechmann I Becher B Luhmann HJ Waisman A Kuhlmann CR Cellular mechanisms of IL-17-induced blood–brain barrier disruption FASEB J 2010 24 1023 1034 10.1096/fj.09-141978 19940258

[B55] Li J Wei GH Huang H Lan YP Liu B Liu H Zhang W Zuo YX Nerve injury-related autoimmunity activation leads to chronic inflammation and chronic neuropathic pain Anesthesiology 2013 118 416 429 10.1097/ALN.0b013e31827d4b82 23340353

[B56] Jovanovic DV Martel-Pelletier J Di Battista JA Mineau F Jolicoeur FC Benderdour M Pelletier JP Stimulation of 92-kd gelatinase (matrix metalloproteinase 9) production by interleukin-17 in human monocyte/macrophages: a possible role in rheumatoid arthritis Arthritis Rheum 2000 43 1134 1144 10.1002/1529-0131(200005)43:5<1134::AID-ANR24>3.0.CO;2-# 10817568

[B57] Prause O Bozinovski S Anderson GP Linden A Increased matrix metalloproteinase-9 concentration and activity after stimulation with interleukin-17 in mouse airways Thorax 2004 59 313 317 10.1136/thx.2003.008854 15047951 PMC1763825

[B58] Ansselin AD Pollard JD Immunopathological factors in peripheral nerve allograft rejection: quantification of lymphocyte invasion and major histocompatibility complex expression J Neurol Sci 1990 96 75 88 10.1016/0022-510X(90)90058-U 2351988

[B59] Argall KG Armati PJ King NJ Douglas MW The effects of West Nile virus on major histocompatibility complex class I and II molecule expression by Lewis rat Schwann cells in vitro J Neuroimmunol 1991 35 273 284 10.1016/0165-5728(91)90181-6 1955569

[B60] Armati PJ Pollard JD Gatenby P Rat and human Schwann cells in vitro can synthesize and express MHC molecules Muscle Nerve 1990 13 106 116 10.1002/mus.880130204 2107399

[B61] Bergsteinsdottir K Kingston A Jessen KR Rat Schwann cells can be induced to express major histocompatibility complex class II molecules in vivo J Neurocytol 1992 21 382 390 10.1007/BF01191706 1607881

[B62] Duan RS Jin T Yang X Mix E Adem A Zhu J Apolipoprotein E deficiency enhances the antigen-presenting capacity of Schwann cells Glia 2007 55 772 776 10.1002/glia.20498 17357152

[B63] Gold R Toyka KV Hartung HP Synergistic effect of IFN-gamma and TNF-alpha on expression of immune molecules and antigen presentation by Schwann cells Cell Immunol 1995 165 65 70 10.1006/cimm.1995.1187 7671325

[B64] Kingston AE Bergsteinsdottir K Jessen KR Van der Meide PH Colston MJ Mirsky R Schwann cells co-cultured with stimulated T cells and antigen express major histocompatibility complex (MHC) class II determinants without interferon-gamma pretreatment: synergistic effects of interferon-gamma and tumor necrosis factor on MHC class II induction Eur J Immunol 1989 19 177 183 10.1002/eji.1830190128 2493382

[B65] Lilje O Armati PJ The distribution and abundance of MHC and ICAM-1 on Schwann cells in vitro J Neuroimmunol 1997 77 75 84 10.1016/S0165-5728(97)00063-5 9209271

[B66] Lisak RP Bealmear B Differences in the capacity of gamma-interferons from different species to induce class I and II major histocompatibility complex antigens on neonatal rat Schwann cells in vitro Pathobiology 1992 60 322 329 10.1159/000163743 1290590

[B67] Samuel NM Jessen KR Grange JM Mirsky R Gamma interferon, but not Mycobacterium leprae, induces major histocompatibility class II antigens on cultured rat Schwann cells J Neurocytol 1987 16 281 287 10.1007/BF01795311 3114433

[B68] Spierings E de Boer T Wieles B Adams LB Marani E Ottenhoff TH Mycobacterium leprae-specific, HLA class II-restricted killing of human Schwann cells by CD4+ Th1 cells: a novel immunopathogenic mechanism of nerve damage in leprosy J Immunol 2001 166 5883 5888 11342602 10.4049/jimmunol.166.10.5883

[B69] Tsai CP Pollard JD Armati PJ Interferon-gamma inhibition suppresses experimental allergic neuritis: modulation of major histocompatibility complex expression of Schwann cells in vitro J Neuroimmunol 1991 31 133 145 10.1016/0165-5728(91)90019-4 1899426

[B70] Tsuyuki Y Fujimaki H Hikawa N Fujita K Nagata T Minami M IFN-gamma induces coordinate expression of MHC class I-mediated antigen presentation machinery molecules in adult mouse Schwann cells Neuroreport 1998 9 2071 2075 10.1097/00001756-199806220-00029 9674595

